# Petrified Ear in Adrenal Insufficiency: Systematic Literature Review

**DOI:** 10.3390/jcm14196870

**Published:** 2025-09-28

**Authors:** Elisa Jörg, Gregorio P. Milani, Sebastiano A. G. Lava, Mario G. Bianchetti, Gabriel Bronz, Pietro B. Faré, Maristella Santi

**Affiliations:** 1 Family Medicine Institute, Faculty of Biomedical Sciences, Università della Svizzera Italiana, 6900 Lugano, Switzerland; elisajoerg.8@gmail.com; 2 Pediatric Unit, Fondazione IRCCS Ca’ Granda Ospedale Maggiore Policlinico, 20122 Milan, Italy; milani.gregoriop@gmail.com; 3 Department of Clinical Sciences and Community Health, Università Degli Studi di Milano, 20122 Milan, Italy; 4 Pediatric Cardiology Unit, Department of Pediatrics, Centre Hospitalier Universitaire Vaudois and University of Lausanne, 1005 Lausanne, Switzerland; webmaster@sebastianolava.ch; 5 Pediatric Institute of Southern Switzerland, Ente Ospedaliero Cantonale, 6500 Bellinzona, Switzerland; 6 Department of Internal Medicine, Regional Hospital of Locarno, Ente Ospedaliero Cantonale, 6600 Locarno, Switzerland; gabriel.bronz@eoc.ch (G.B.); farepietro@gmail.com (P.B.F.); 7 Department of Pediatrics, Fribourg Hospital HFR, 1708 Fribourg, Switzerland; maristella.santi@h-fr.ch; 8 Faculty of Science and Medicine, University of Fribourg, 1700 Fribourg, Switzerland

**Keywords:** Addison’s disease, adrenal insufficiency, ossification of the auricle, petrified ear

## Abstract

**Background**: Adrenal insufficiency has been recognized as a condition linked to petrified ear. To further explore this issue, we conducted a review of the literature. **Methods**: The study was performed in accordance with the Preferred Reporting Items for Systematic Reviews and Meta-Analyses guidelines after pre-registration. Excerpta Medica, the National Library of Medicine, and Web of Science databases without language restrictions were used. Inclusion criteria comprised adrenal insufficiency and the presence of petrified ear. Data extraction included demographics, clinical and laboratory features, and outcome. **Results**: Thirty-six reports were identified, encompassing 40 cases: 38 males and 2 females, aged between 22 and 79 years. All cases exhibited bilateral petrified ears, with 18 cases of primary adrenal insufficiency and 20 cases of central insufficiency. The type of adrenal insufficiency was not specified in two cases. Sixteen patients had concurrent endocrine disorders. In primary adrenal insufficiency, petrified ear was typically (N = 13; 72%) detected two or more years after the endocrine diagnosis. In central adrenal insufficiency, auricular calcification was identified either prior to or at the time (N = 17; 85%) of the endocrine diagnosis. Petrified ear was never associated with hearing impairment and never improved with therapy. **Conclusions**: In adrenal insufficiency, petrified ear is always bilateral, affects adult males, occurs in both primary and central forms of the disease and does not improve on therapy. In primary insufficiency, this feature is mostly observed two years after the endocrine diagnosis, whereas in central cases, it is identified before or concurrently with the endocrine diagnosis.

## 1. Introduction

In 1866, the Czech anatomist Vincenz Bochdalek (1801–1883) reported that the auricle may occasionally present as rigid, hard, thick, and with a rock-like consistency [1]. This typically asymptomatic yet remarkable and uncommon clinical finding is referred to as petrified ear, petrified auricle, or stony ear, and may result from calcification, ossification, or both [2].

Repeated physical trauma to the auricle, prolonged exposure to cold (with or without frostbite), chronic infections, and endocrine diseases such as hypothyroidism and acromegaly have been proposed as potential contributing factors [3,4,5]. Of particular note, adrenal insufficiency has been recognized since at least 1954 as a condition associated with petrified ear [6], a feature that has since been reported exclusively in isolated case reports, thereby highlighting both its rarity and diagnostic significance.

Textbooks and authoritative reviews generally describe hyperpigmentation affecting sun-exposed areas, palmar creases, flexural surfaces, friction sites, recent scars, genital skin, and mucous membranes as hallmark dermatological and mucosal features of primary adrenal insufficiency [7]. By contrast, the occurrence of petrified ear in the setting of adrenal insufficiency is either not mentioned at all or only marginally addressed in these sources [7,8,9], despite its potential clinical relevance. Case reports undoubtedly offer valuable insights into rare and unusual manifestations, yet it is only through their systematic aggregation and critical evaluation that consistent patterns can be recognized, clinical characteristics more clearly delineated, and possible pathophysiological mechanisms revealed. Motivated by these considerations, and further stimulated by our direct encounter with a male patient simultaneously presenting adrenal insufficiency and petrified ear, we undertook a systematic review of the literature. This effort aimed not only to collect clinical observations but also to provide a structured framework for understanding this uncommon, intriguing association.

## 2. Materials and Methods

### 2.1. Literature Search Strategy

This study was pre-registered with the International Prospective Register of Systematic Reviews (PROSPERO: CRD42025633721) and was conducted in accordance with the guidelines outlined in the 2020 edition of the Preferred Reporting Items for Systematic Reviews and Meta-Analyses. Literature searches were performed in Excerpta Medica, the National Library of Medicine, and Web of Science databases, for articles or letters published since 1954 without language restrictions. Search terms included (“ear” OR “calcification of the ear” OR “auricular cartilage calcification” OR “petrified ears”) AND (“adrenal insufficiency” OR “corticoadrenal insufficiency” OR “Addison’s disease”). Additional references were identified through Google Scholar and bibliographies of retrieved records. The search was conducted in January 2025 and repeated on 31 August 2025. After a preliminary screening based on titles and abstracts, the full texts of the selected studies were reviewed for eligibility.

### 2.2. Selection Criteria—Data Extraction

Original reports documenting otherwise unexplained cases of petrified ear in patients of all ages diagnosed with primary or central adrenal insufficiency were considered. Cases published solely as abstracts were disregarded. The following five data sets were extracted for each individual with petrified ear linked to adrenal insufficiency using a structured piloted form: (1) demographics and temporal relationship between diagnosis of adrenal insufficiency and recognition of petrified ear (clinical sign detected before endocrine diagnosis by ≥2 years, clinical sign and endocrine diagnosis detected concurrently, clinical sign detected ≥2 years after endocrine diagnosis), (2) laboratory data supporting the classification of adrenal insufficiency as primary or central, (3) the presence of other concomitant endocrine disorders (such as diabetes mellitus, growth hormone deficiency, hyperprolactinemia, hypogonadism, or hypothyroidism), (4) blood levels of “phosphocalcemic factors” such as calcium, inorganic phosphate, parathyroid hormone, and vitamin D [10], (5) investigations, in addition to clinical examination, documenting the petrified ear, its clinical course, and the presence of hearing impairment.

### 2.3. Reporting Thoroughness—Statistics

For each individual case, the thoroughness of reporting for the five specified data sets was assessed on a scale of 0, 1, or 2. Based on the total score, the overall thoroughness of reporting for each case was categorized as excellent (9 or 10), good (7 or 8), or satisfactory (5 to 7), according to our standard procedure [11,12].

Two authors independently and in duplicate conducted the literature search, selected reports for inclusion, extracted the data using a structured, pilot-tested paper form, and assessed the thoroughness of each reported case. Any discrepancies were resolved through discussion, with input from a senior author when necessary. One author then entered the data into a spreadsheet, while another cross-checked their accuracy.

Pairwise deletion was applied to address missing data [13]. Categorical variables are presented as absolute counts and, where appropriate, also expressed as percentages. Percentages were rounded to the nearest whole number when the value was equal to or greater than 10, whereas for values below 10, they were rounded to one decimal place. Binary data were analyzed using Fisher’s exact test, while ordered data were evaluated with the Wilcoxon–Mann–Whitney rank-sum test [14]. Continuous variables are presented as medians with interquartile ranges (≥5 cases) or as individual values (<5 cases) and were assessed using the Kruskal–Wallis test by ranks [14]. A two-sided *p*-value threshold of <0.05 was used to determine statistical significance. GraphPad Prism version 10.4.1 (GraphPad Software, San Diego, CA, USA) was used for all analyses.

## 3. Results

### 3.1. Search Results

**Figure 1:**
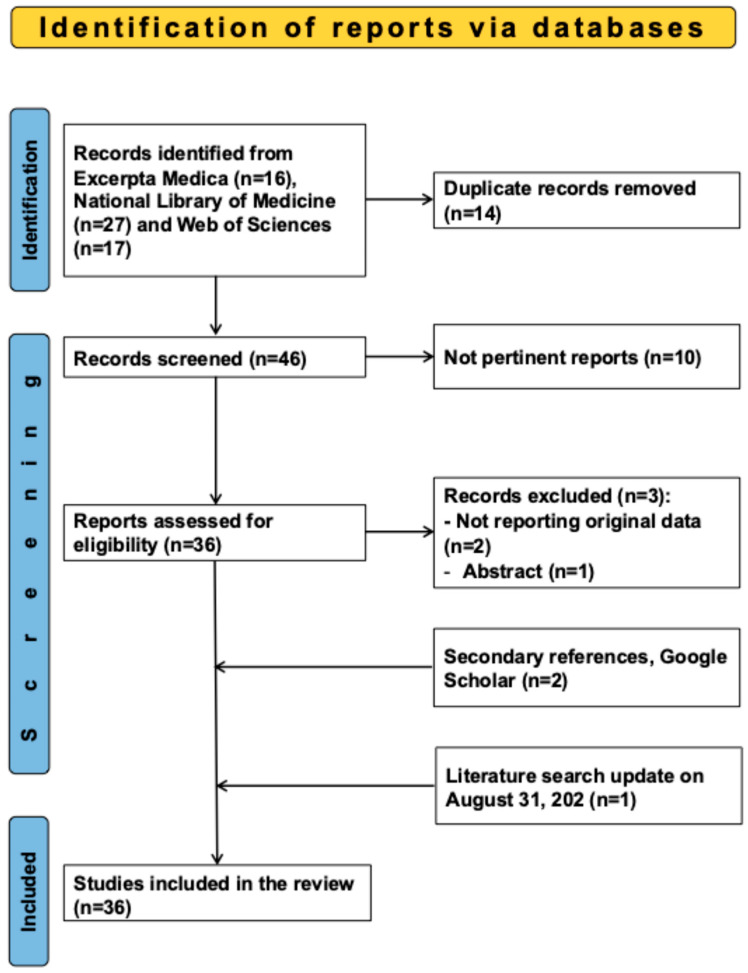
Study Selection Flowchart.

The study flowchart is shown in Figure 1.

For the final analysis, we reviewed 36 reports published since 1955 that documented auricular calcifications linked to adrenal insufficiency [15,16,17,18,19,20,21,22,23,24,25,26,27,28,29,30,31,32,33,34,35,36,37,38,39,40,41,42,43,44,45,46,47,48,49,50]. The articles were written in English (N = 28), German (N = 4), Spanish (N = 3), and French (N = 1). They came from the following continents: 17 from Europe (Germany, N = 3; Spain, N = 3; Switzerland, N = 3; Italy, N = 2; France, N = 1; Greece, N = 1; The Netherlands, N = 1; Portugal, N = 1; Romania, N = 1; United Kingdom, N = 1), 11 from Asia (Israel, N = 4; Japan, N = 3; India, N = 2; Taiwan, N = 1; Türkiye, N = 1), and 8 from America (United States, N = 6; Canada, N = 1, Chile, N = 1). The 36 selected articles documented 40 individuals with petrified ear linked to adrenal insufficiency. Reporting thoroughness was assessed as excellent, good, and satisfactory in 17 (43%), 17 (43%), and 6 (15%) cases, respectively.

### 3.2. Findings

The diagnosis of petrified was established clinically in all 40 cases. Imaging studies confirmed the diagnosis in 35 cases, while a biopsy provided additional confirmation in 7 cases.

The characteristics of the 40 patients appear in Table 1.

Petrified ear was always bilateral and was recognized exclusively in adults, with 95% of cases being male. Primary and central adrenal insufficiency were represented in a similar manner (this classification was not possible in two cases).

Adrenal insufficiency was associated with at least one more endocrine condition in 40% of cases. The overall prevalence (*p* = 0.0070) of associated endocrine conditions, especially hypogonadism (*p* = 0.0366), was significantly higher in central than in primary cases. The prevalence of hypothyroidism, diabetes mellitus, growth hormone deficiency, and hyperprolactinemia was similar in cases of primary and central adrenal insufficiency.

In primary adrenal insufficiency, petrified was typically (72%) detected two or more years after the endocrine diagnosis. In contrast, in the majority (85%) of central adrenal insufficiency cases, petrified ear was identified either prior to or at the time of the endocrine diagnosis. This difference was statistically significant (*p* = 0.0381). Hypercalcemia, hyperphosphatemia, elevated parathyroid hormone or vitamin D levels were almost never described.

Finally, petrified ear was never associated with hearing impairment and did not improve with replacement therapy.

## 4. Discussion

This systematic review of the literature on petrified ear in adrenal insufficiency indicates that this clinical feature is always bilateral, almost uniquely affects adult males, occurs in both primary and central forms of the disease, is often associated with other endocrine diseases, and does not improve on therapy. In primary adrenal insufficiency, the feature is typically recognized two or more years after the endocrine diagnosis. In contrast, in cases with central adrenal insufficiency, the feature is generally identified either prior to or at the time of the endocrine diagnosis.

The mechanisms driving petrified ear in adrenal insufficiency are not well understood. However, it is mostly thought to be attributable to hypercalcemia, a recognized electrolyte disturbance in adrenal insufficiency [7,8,9,10]. Parathyroid hormone and 1,25-dihydroxyvitamin D are generally reduced in hypercalcemic individuals with adrenal insufficiency, indicating a suppressed parathyroid hormone–vitamin D axis [7,8,9,10]. In this context, hypercalcemia is currently thought to result from increased bone resorption that occurs independently of parathyroid hormone, and from fluid volume depletion [10,51,52]. In patients with petrified ear linked to adrenal insufficiency, hypercalcemia had virtually never been documented. However, it is plausible that petrified ear might be linked to prior episodes of hypercalcemia. This speculation is supported by the fact that chronic hypercalcemic disorders such as primary hyperparathyroidism and sarcoidosis are recognized triggers of petrified ear [51,52]. Excess adrenocorticotropic hormone is a well-recognized cause of tissue calcification [5,53]. However, this mechanism is unlikely to explain adrenal insufficiency-associated petrified ear, as the condition has been observed in both primary and central forms [7,8,9].

Petrified ear, whether associated with adrenal disorders or not, affects almost exclusively males [3,4,5]. Although the underlying mechanisms remain unknown, it is conceivable, though not demonstrated, that estrogens may play a protective role [54].

It is plausible that the pathological process underlying petrified ear may also involve the middle or inner ear, thereby leading to hearing impairment. However, in none of the cases of petrified ear included in our analysis was hearing impairment documented. It is nevertheless likely that this aspect has not always been investigated. We therefore recommend that this possibility be considered in patients with petrified ear.

While the hypothalamic–pituitary–adrenal axis is altered in both primary and central adrenal insufficiency, the renin-angiotensin II-aldosterone axis and the adrenal medullary function are affected only in the primary form [7,8,9]. Both types of adrenal insufficiency present with chronic, non-specific features like fatigue, weight loss, gastrointestinal discomfort, and joint or muscle pain, but water and electrolyte disturbances mainly occur in primary adrenal insufficiency. As a result, diagnosis is typically quicker in the primary form [7,8,9]. This may be why petrified ears are often detected at diagnosis in central adrenal insufficiency.

The prevalence of petrified ears in adrenal insufficiency is unknown, but likely very low considering that only 40 cases have been reported since 1955. According to a report published in 1954, the existence of petrified ear would have been observed in 6 out of 120 patients with adrenal insufficiency [6]. To the best of our knowledge, the prevalence of this sign among individuals with adrenal insufficiency has never been addressed in the literature since. Because adrenal insufficiency is nowadays diagnosed earlier than 70 years ago, we suppose that this feature might be even less common today.

Besides petrified ear, petrified auricle, and stony ear, the term auricular cartilage calcification is also used. We recommend avoiding “cartilage” and favoring auricular calcification, although all these terms inadequately capture the associated thickening of the auricle.

Rediscovering the value of bedside skills is crucial, as they remain central to timely and accurate diagnosis despite rapid technological progress. In medical practice, technology has advanced tremendously, often reducing the emphasis on skills like history taking and physical examination. Nevertheless, inadequacies of history taking or examination, often oversights, remain recognized contributors to medical errors [55,56]. Because the presentation of adrenal insufficiency is nonspecific, this diagnosis is often delayed [7,8,9]. The findings of this literature review indicate that petrified ear, along with hyperpigmentation, constitutes a simple and noteworthy clinical sign. On one hand, petrified ear should be sought whenever adrenal insufficiency is suspected; on the other hand, bilaterally petrified ears with no evident cause should prompt consideration of adrenal insufficiency.

Future research may first involve a structured survey among endocrinologists to better estimate how frequently petrified ear occurs and to what extent it is recognized in routine clinical practice. Another promising line of investigation would be to assess whether comparable processes of calcification and ossification are present in other anatomical sites, thereby shedding light on possible systemic mechanisms underlying this condition [6]. Finally, in vitro studies of cartilaginous tissue could provide important mechanistic insights by comparing cellular and structural changes under different hormonal environments, particularly in the presence or absence of cortisol and estrogens [54]. Considering that petrified ear almost exclusively affects males, such experiments would be especially relevant to clarify the role of sex hormones in the pathogenesis and potential protection afforded by estrogens [54].

This systematic review has both limitations and merits. The main limitations are the small sample size and incomplete reporting in some cases, which may reduce the generalizability of the results. Furthermore, diagnostic techniques used until the 1980s were often relatively rudimentary. An additional drawback is that the prevalence of this sign remains unknown. The merits include its pre-registration, a thorough case selection methodology, and the use of multiple languages. Additionally, the study highlights a rarely discussed feature of adrenal insufficiency, provides some new insights on the condition, and presents a review spanning seven decades.

## 5. Conclusions

This review identifies petrified ear as a rare feature of adrenal insufficiency, highlighting its earlier detection in central compared to primary forms. The finding that this clinical feature does not improve with therapy underscores its potential as a distinctive and lasting diagnostic marker. By firmly linking this uncommon sign to adrenal insufficiency, the study provides clinicians with a simple yet valuable clue that can meaningfully aid in diagnosis.

## Figures and Tables

**Table 1:** Characteristics of 40 patients, 22 to 79 years of age, with bilateral petrified ear associated with adrenal insufficiency. Data are presented as frequency (and sometimes also as percentage) or as median and interquartile range (individual values if <5 cases).

		Adrenal Insufficiency				*p*-Value
		All	Primary	Central	Unclassified	
**N**		40	18	20	2	
**Demographics**						
	Males:females	38:2	18:0	18:2	2:0	0.5385
	Age ^∆^, years	56 (44–67)	55 (46–70)	49 (37–62)	60, 70	0.1917
**Concomitant endocrine conditions**		16 (40)	3 (17)	11 (55)	2 (100)	0.0070
	Hypothyroidism	11 (28)	3 (17)	7 (35)	1 (50)	0.2672
	Type 2 diabetes mellitus	1 (2.5)	0	0	1 (50)	>0.9999
	Hypogonadism	5 (13)	0	5 (25)	0	0.0366
	Growth hormone deficiency	3 (7.5)	0	3 (15)	0	0.0734
	Hyperprolactinemia	3 (7.5)	0	3 (15)	0	0.0734
**Temporal relationship with adrenal disease diagnosis**						0.0381
	Calcification before by ≥2 years	4 (10)	2 (11)	1 (5.0)	1 (50)	
	Calcification and adrenal disease concurrently	19 (48)	3 (17)	16 (80)	0	
	Calcification after by ≥2 years	17 (43)	13 (72)	3 (15)	1 (50)	
**Phosphocalcemic factors**						
	Hypercalcemia	1 (2.5)	0	1 (5.0)	0	>0.9999
	Hyperphosphatemia	1 (2.5)	0	1 (5.0)	0	>0.9999
	Parathyroid hormone level increased	0	0	0	0	>0.9999
	Vitamin D level increased	0	0	0	0	>0.9999

^∆^ at diagnosis of petrified ear.

## Data Availability

Data sharing does not apply to this article, as no new data were generated during this study.
